# Aspects of varicella-zoster infection in children

**DOI:** 10.1186/1471-2334-13-S1-P93

**Published:** 2013-12-16

**Authors:** Consuela Marcaş, Ana Maria Iancu, Ilie Maria Margareta

**Affiliations:** 1 Clinical Hospital of Infectious Diseases, Constanța, Romania

## Background

Herpes zoster is an infectious and contagious disease caused by the reactivation of latent varicella-zoster virus. Shingles occurs sporadically and usually in adults and the elderly (over 90% of the cases occur after the age of 20 years). It occurs rarely in children (5%).

## Methods

We studied the cases of herpes zoster which were admitted in the hospital in the last 12 months (June 2012- July 2013) in the Pediatric Department.

## Results

Seven children and infants were admitted with a of diagnosis herpes zoster based on clinical judgment. Their ages were between 4 months and 5 years, three of them being infants. None of the children had prior exposure to varicella-zoster virus during gestation or early infancy.

One case presented ocular involvement, 6 patients had thoracic form. One of the infants presented a spread of infection to more dermatomes. Two patients presented complications (cardiac and digestive). None of the children were not vaccinated.

## Conclusion

Compared to previous years, we noticed a decreasing age of children and infants with this condition. All children and infants were without medical history of chickenpox. None of the mothers had presented chickenpox in the second or third trimester of pregnancy.

